# Desire to Avoid Pregnancy scale: clinical considerations and comparison with other questions about pregnancy preferences

**DOI:** 10.1136/bmjsrh-2022-201750

**Published:** 2023-01-30

**Authors:** Jennifer Anne Hall, Geraldine Barrett, Judith M Stephenson, Natalie Lois Edelman, Corinne Rocca

**Affiliations:** 1 Research Department of Reproductive Health, UCL Institute for Women's Health, London, UK; 2 School of Sport & Health Sciences, University of Brighton, Brighton, UK; 3 Primary Care & Public Health, Brighton and Sussex Medical School, Brighton, UK; 4 Advancing New Standards in Reproductive Health (ANSIRH), Department of Obstetrics, Gynecology and Reproductive Sciences, University of California, San Francisco (UCSF) School of Medicine, Oakland, San Francisco, California, USA

**Keywords:** family planning services, Reproductive Health, Reproductive Health Services

## Abstract

**Background:**

Clinicians and women of reproductive age would benefit from a reliable way to identify who is likely to become pregnant in the next year, in order to direct health advice. The 14-item Desire to Avoid Pregnancy (DAP) scale is predictive of pregnancy; this paper compares it with other ways of assessing pregnancy preferences to shortlist options for clinical implementation.

**Methods:**

A cohort of 994 UK women of reproductive age completed the DAP and other questions about pregnancy preferences, including the Attitude towards Potential Pregnancy Scale (APPS), at baseline and reported on pregnancies quarterly for a year. For each question, DAP item and combinations of DAP items, we examined the predictive ability, sensitivity, specificity, area under the receiver operating curve (AUROC), and positive and negative predictive values.

**Results:**

The AUROCs and predictive ability of the APPS and DAP single items were weaker than the full DAP, though all except one had acceptable AUROCs (>0.7). The most predictive individual DAP item was ‘It would be a good thing for me if I became pregnant in the next 3 months’, where women who strongly agreed had a 66.7% chance of pregnancy within 12 months and the AUROC was acceptable (0.77).

**Conclusion:**

We recommend exploring the acceptability to women and healthcare professionals of asking a single DAP item (‘It would be a good thing for me if I became pregnant in the next 3 months’), possibly in combination with additional DAP items. This will help to guide service provision to support reproductive preferences.

**WHAT IS ALREADY KNOWN ON THIS TOPIC**

- Clinicians do not currently have a valid and reliable way of asking women about their pregnancy preferences.

**WHAT THIS STUDY ADDS**

- A single item from the Desire to Avoid Pregnancy (DAP) scale is effective at identifying who is likely to become pregnant in the next year; other questions, including the Attitude towards Potential Pregnancy Scale, are less discriminative but less burdensome than the full DAP Scale.

**HOW THIS STUDY MIGHT AFFECT RESEARCH, PRACTICE OR POLICY**

- The acceptability to women and health professionals of different ways of asking women about their pregnancy preferences in healthcare settings should be explored.

## Introduction

While there are a multitude of measures and screening tools available to predict pregnancy-related conditions, such as pre-eclampsia^1^ or gestational diabetes,^2^ there are no valid and reliable clinical tools to identify women who are likely to become pregnant (where we refer to ‘women’ this should be taken to include people who do not identify as women but who have the capability to become pregnant).^1^ Such a tool would have great utility for clinicians working with women of reproductive age as it could be used to guide discussions on either preparation for pregnancy or contraceptive options, depending on if or when a pregnancy might be desired. This would simultaneously help to prevent unintended pregnancies and improve pregnancy outcomes and long-term health.^3^ Research shows that most clinicians see the value of such discussions^4–7^ but highlights the need for further evidence on the ‘validity, appropriate use, and integration into healthcare systems’.^4^

The Desire to Avoid Pregnancy (DAP) scale is a psychometrically validated measure of pregnancy preferences that was developed in the USA in 2019,^8^ and validated in the UK in 2022.^9^ It was shown to be highly predictive of pregnancy, with women with the lowest DAP score having an 80% chance of becoming pregnant within 12 months.^9^ Subsequently, the sensitivity and specificity of the DAP have been described (0.78 and 0.81, respectively, at a cut-point <2)^10^ and further analysis has shown how DAP score is associated with sociodemographic factors,^10^ findings which are in keeping with the literature on pregnancy preferences.^11–13^

While there may be ways of incorporating the DAP scale into clinical practice, for example, preconsultation digital use or self-completion, a tool with 14 items is likely to be impractically lengthy in many clinical consultations. Use of selected items from the DAP, or other questions, may provide a brief and viable alternative if they distinguish sufficiently between who will and who will not become pregnant. There are limited options in terms of pregnancy intention screening tools with evidence of their performance. The only other measure with any psychometric evaluation is the five-question Attitude toward Potential Pregnancy Scale (APPS),^14^ though there is only one published use of it.^15^ Alternatively, ‘One Key Question’ (OKQ), which is a question not a measure, asks ‘Would you like to become pregnant in the next year?’ and is packaged with training and protocols.^16^ There is mixed evidence on the acceptability and effectiveness of the OKQ.^17–20^ There are no data on the performance or predictive ability of these alternatives and, until the DAP scale, there had not been a ‘gold standard’ to compare them to.

The aims of this paper are to: (1) evaluate the predictive ability, sensitivity, specificity, area under the receiver operating curve (AUROC) and positive and negative predictive values (PPV and NPV) of different methods of assessing pregnancy preferences and (2) examine the same criteria for individual DAP items and selected combinations of DAP items. Using the findings of these two aims, options are shortlisted for consideration for clinical implementation.

## Methods

### Sample

We analysed data from a cohort of 994 non-pregnant women, aged 15 and over, who were recruited in the UK in October 2018 and followed up every 3 months for 1 year. The full details of recruitment and participation are described elsewhere,^9^ but in brief, people who self-reported as female, were premenopausal and not sterilised were recruited though advertising at a school, a university, a sexual health clinic and a pregnancy termination clinic, as well as through online recruitment via both paid advertisements (Instagram and Facebook) and sharing through networks.^9^ The survey was programmed in RedCap^21 22^ and included the DAP scale, other questions about pregnancy preferences and sociodemographics. At each quarterly follow-up, participants were asked whether they were currently pregnant or had been pregnant since the last survey. Almost 90% (831/929) of eligible women completed follow-up at 12 months; those who were lost to follow-up were not significantly different by age, relationship status, ethnicity, baseline DAP score or number of children from those who did (suggesting that there is no selection bias in the lost to follow-up).^10^

### Measures

#### Outcome

We created a binary variable of any incident pregnancy between baseline and 12 months. Given low attrition and to ease interpretation, we included participants in pregnancy denominators until they were lost to follow-up and report percentages rather than rates.

#### Attitude towards Potential Pregnancy Scale

The APPS is a five-item measure of a woman’s emotional outlook regarding a potential pregnancy (table 1).^14^ Each item is scored one-to-five on a visual anchored scale and summed to give an overall score from five to 25; higher scores represent a more positive attitude towards pregnancy. The APPS had a Cronbach’s alpha of 0.86 among 130 participants in the USA but has not been examined in the UK.

**Table 1:** Wording of questions about preferences regarding future pregnancy

Question wording	Response options	Domain
DAP (with label for reference in text)		
I wouldn’t mind if I became pregnant in the next 3 months Pregnant: wouldn’t mind	0 Strongly agree—4 strongly disagree	1
It would be a good thing for me if I became pregnant in the next 3 months Pregnant: good thing for me	0 Strongly agree—4 strongly disagree	1
Thinking about becoming pregnant in the next 3 months makes me feel unhappy. Pregnant: unhappy	4 Strongly agree—0 strongly disagree	2
Thinking about becoming pregnant in the next 3 months makes me feel excited. Pregnant: excited	0 Strongly agree—4 strongly disagree	2
Becoming pregnant in the next 3 months would bring me closer to my main partner Pregnant: closer to partner	0 Strongly agree—4 strongly disagree	3
I want to have a baby within the next year. Baby: want	0 Strongly agree—4 strongly disagree	1
If I had a baby in the next year, it would be bad for my life. Baby: bad for life	4 Strongly agree—0 strongly disagree	1
It would be a positive addition to my life to have a baby in the next year. Baby: positive addition to life	0 Strongly agree—4 strongly disagree	1
It would be the end of the world for me to have a baby in the next year Baby: end of the world for me	4 Strongly agree—0 strongly disagree	1
Thinking about having a baby within the next year makes me smile Baby: makes me smile	0 Strongly agree—4 strongly disagree	2
Thinking about having a baby within the next year make me feel stressed out. Baby: stressed out	4 Strongly agree—0 strongly disagree	2
I would feel a loss of freedom if I had a baby in the next year. Baby: loss of freedom	4 Strongly agree—0 strongly disagree	3
If I had a baby in the next year, it would be hard for me to manage raising the child. Baby: hard for me to manage	4 Strongly agree—0 strongly disagree	3
I would worry that having a baby in the next year would make it harder for me to achieve other things in my life. Baby: hard to achieve other things	4 Strongly agree—0 strongly disagree	3
APPS		Not applicable
How much do you want to be pregnant now?	1 Not at all—5 Very much	
How important is it to you to avoid become pregnant now?	5 Not at all important—1 Very important	
How worried would you be if you were pregnant now?	5 Not worried at all—1 Very worried	
How upset would you be if you were pregnant now?	5 Not at all upset—1 Very upset	
How happy would you be if you were pregnant now?	1 Very unhappy—5 Very happy	
Feeling question		
Overall, when I think about become pregnant in the next 3 months, I feel:	1 Mostly positive	
	2 More positive than negative	
	3 In the middle between positive and negative	
	4 I don’t feel strongly one way or the other	
	5 More negative than positive	
	6 Most negative	
Trying question		
Are you currently trying to get pregnant?	1 Yes	
Thinking question		
Are you thinking about trying to get pregnant in the next year?	2 Yes	

Domains: 1 = Cognitive desires and preferences, 2 = Affective feelings and attitudes, 3 = Anticipated practical consequences. Further information on the DAP wording and instructions for use are available here: https://www.ansirh.org/sites/default/files/publications/files/desire_to_avoid_pregnancy_scale_english_6_22_20.pdf.

APPS, Attitude towards Potential Pregnancy Scale; DAP, Desire to Avoid Pregnancy.

#### Other pregnancy preference questions

We compared the DAP score with three other single questions asking about pregnancy preferences that were available when the study was designed in 2018 (table 1). The ‘feelings question’ was used in the ADAPT study^23^; the ‘trying’ and ‘thinking’ questions are ones that clinicians in the UK have told us are the kind of questions they currently use. We did not use OKQ as it is a proprietary tool that is unlikely to be available for widespread implementation in the UK, but the ‘thinking’ question is similar.

#### DAP Scale

The DAP scale is a psychometrically validated measure covering three conceptual domains: (1) cognitive desires and preferences; (2) affective feelings and attitudes; and (3) anticipated practical consequences (table 1).^8^ Each item uses a Likert scale, scored 0–4, to ask women how much they agree or disagree with a statement about either becoming pregnant in the next 3 months or having a baby in the next year. Responses are averaged, producing a total score between 0 and 4; higher scores represent a higher DAP. It was found to be highly acceptable in the UK evaluation.^9^

### Plan of analysis

#### Aim 1: evaluating the performance of other methods of assessing pregnancy preferences

We assessed the APPS’s reliability (internal consistency) with Cronbach’s alpha (>0.7 considered acceptable)^24^ and checked that all item-rest correlations were >0.2.^25^ We examined construct (structural) validity via principal components analysis and considered the scale structurally valid if all items loaded on to one component with an Eigenvalue >1.^26^

To compare the assessment of pregnancy preferences by the DAP versus the APPS and the three individual questions (‘feeling’, ‘trying’ and ‘thinking’), we examined the relationships among them. As the DAP and the APPS yield continuous scores, we used the Pearson correlation coefficient to examine the strength of association, expecting a negative correlation (ie, as DAP score increases, APPS score decreases), considering a strong correlation to be lower than −0.7. This also served as a test of the APPS’s concurrent validity. The range of DAP scores within, and distribution of DAP scores across, the response options of each question was examined. The Kruskal-Wallis test was used to assess whether differences in median DAP score across response options within each question were significant.

To investigate how well each approach predicted actual pregnancy, we modelled the probability of pregnancy using logistic regression models and examined the sensitivity, specificity, AUROC, PPV and NPV, using the Youden index suggested cut-point.^27^ The AUROC represents the tool’s ability to identify who will become pregnant. An AUROC of 0.7–0.8 was considered acceptable, 0.8–0.9 excellent and >0.9 outstanding.^28^

#### Aim 2: examination of the performance of individual and combinations of DAP items

We explored the predictive ability of each individual DAP item, as well as selected combinations of items, using logistic regression, with incident pregnancy as the outcome. The item combinations were designed to ensure coverage of the three conceptual domains, positively and negatively framed items, different time frames and use of ‘pregnancy’ and ‘baby’, as well as the predictive ability of the items. The combinations were cross-validated by developing them on one half of the data (created with a random split) and tested on the other half to avoid overfitting and give a more accurate estimate of how the questions perform outside the data they were developed from.^29 30^ The results from the testing data are presented. The predictive ability, sensitivity, specificity, AUROC, PPV and NPV of the selected items/combinations were calculated (using the Youden index suggested cut-point)^27^ and compared with the total DAP scale.

To inform which question(s) provided the best balance between brevity and predictive ability, the performance of the single questions, the APPS and selected DAP items/combinations were compared using the predictive ability, AUROC and number of items/questions, to make recommendations on which item/question(s) should be taken forward for further consideration.

### Patient and public involvement

We undertook public involvement in the development of our overall programme of research on pregnancy planning, the P3 Study, by discussing and refining ideas during the grant application stage with an existing patient and public involvement (PPI) group within our team. Findings were frequently discussed with the P3 Study’s PPI group to inform the next steps and the group is currently planning our wider dissemination.

## Results

### Sample

As previously described,^9^ the baseline cohort of 994 women were aged 15–50 years (median=31, IQR=23–36, mean=29.7). The sample was fairly evenly distributed by age, with 28% aged 15–24 years, 36% aged 25–34 and 31% aged over 35 (4% did not give an age). Most (82%) were in a relationship and 82% described themselves as heterosexual. The majority (84%) identified as white; 6.9% as Asian, 2.6% as Mixed, 2.4% as Black and 0.9% as Other (3.2% missing/preferred not to say). Around one in 10 women did not speak English as their first language. The sample was quite highly educated with 39% having an undergraduate degree and 31% a postgraduate or professional qualification. Fifty-seven per cent had one or more children in their household. The dataset is available in the UCL Research Data Repository.^31^

### Aim 1: evaluating the performance of other methods of assessing pregnancy preferences

The full range of APPS scores^5–25^ was reported in our cohort. The Cronbach’s alpha for the APPS was 0.93, all item-rest correlations were >0.2 and positive, and all items loaded on to one component with an Eigenvalue of 3.87. The Pearson correlation coefficient between the APPS and DAP was −0.893 (p<0.001) showing a strong negative correlation (ie, as a woman’s attitude towards pregnancy becomes more positive, her DAP score reduces.) The median DAP score was statistically significantly different across the response options for the APPS and each of the ‘feeling’, ‘trying’ and ‘thinking’ questions (p values in table 2). For example, women who felt ‘mostly positive’ about pregnancy had a median DAP score of 0.64 (low DAP) whereas women who felt ‘mostly negative’ about pregnancy had a median DAP score of 3.50 (high DAP).

**Table 2:** Relationship with DAP and predicted probability, sensitivity, specificity, AuROC, PPV and NPV of other questions

	n	Median DAP score	IQR	P value	Predicted probability of pregnancy	95% CI	Sensitivity	Specificity	AuROC	PPV	NPV
APPS score*							78%	74%	0.76	36%	95%
5	265	3.64	3.29 to 3.86	<0.001	0.03	0.019 to 0.044					
10	59	2.50	2.29–2.71		0.09	0.063 to 0.107					
15	35	1.71	1.50–2.07		0.21	0.173 to 0.243					
20	13	1.07	0.86–1.50		0.47	0.362 to 0.490					
25	42	0.57	0.36–0.64		0.68	0.594 to 0.761					
Overall, when I think about becoming pregnant in the next 3 months, I feel:											
Mostly positive	117	0.64	0.43–0.93	<0.001	0.56	0.47 to 0.65	75%	76%	0.75	37%	94%
More positive than negative	84	1.29	1.07–1.64		0.37	0.26 to 0.48					
In the middle	107	2.00	1.57–2.36		0.14	0.07 to 0.21					
Don't feel strongly	21	2.14	2.00–2.43		0.07	−0.06 to 0.19					
More negative than positive	264	2.64	2.36–3.00		0.09	0.06 to 0.13					
Mostly negative	395	3.50	3.14–3.79		0.03	0.02 to 0.05					
Are you currently trying to get pregnant?											
Yes	80	0.64	0.36–0.86	<0.001	0.69	0.58 to 0.50	37%	97%	0.67	69%	89%
No	907	2.79	2.07–3.43		0.11	0.09 to 0.13					
Are you thinking about trying to get pregnant in the next year?											
Yes	96	1.21	0.79–1.71	<0.001	0.53	0.42 to 0.63	71%	84%	0.77	34%	96%
Maybe	97	1.86	1.36–2.21		0.15	0.07 to 0.22					
No	707	3.07	2.57–3.57		0.04	0.20 to 0.06					

*APPS score is continuous from 5 to 25, 5 points shown for illustration, therefore, n does not add up to the full sample.

APPS, Attitude towards Potential Pregnancy Scale; AUROC, area under the receiver operating curve; DAP, Desire to Avoid Pregnancy; NPV, negative predictive value; PPV, positive predictive value.

The AUROCs and predictive ability of the APPS and the single questions were weaker than the full DAP (AUROC 0.87, predictive ability 79.4%), though all except the ‘trying’ question had acceptable AUROCs of >0.7.

### Aim 2: evaluating the performance of individual and combinations of DAP items

All DAP items and domains were associated with pregnancy. The best performing individual DAP item in terms of pregnancy prediction was ‘Pregnancy: good thing for me’, where there was a 66.7% chance of pregnancy within 12 months among women who strongly agreed (table 3). At a cut point of 2.5 this item also had an acceptable AUROC (0.77).

**Table 3:** Exploration of the predicted probability, sensitivity, specificity, AuROC, PPV and NPV of selected individual and combined DAP items

Description	Predicted probability of pregnancy at DAP score		Empirical cut-point	Sensitivity	Specificity	AuROC	PPV	NPV	Domains covered	Positive and negatively worded items
	0	4								
Total DAP: All 14 items	79.4%	0.9%	1.96	78.0%	81.0%	0.85	43.0%	95.0%	All	Yes
Single DAP item: Pregnant: good thing for me.	66.7%	2.1%	2.5	68.1%	86.3%	0.77	49.0%	93.3%	1	Positive only
Single DAP item: Baby: end of the world for me.	27.4%	–	0.5	65.3%	77.7%	0.72	36.2%	92.0%	1	Negative only
Two DAP items: Pregnant: good thing for me and Baby: end of the world for me.	71%	0.7%	1.25	65.3%	90.1%	0.78	56.0%	93.1%	1	Yes
Three DAP items: Pregnant: good thing for me, Pregnant: excited, Baby: hard to achieve other things.	83%	2.8%	2.15	62.5%	87.1%	0.75	48.4%	92.3%	All	Yes
Three DAP items: Pregnant: good thing for me, Pregnant: excited, Baby: end of the world for me.	73%	1.2%	1.49	66.7%	87.4%	0.77	50.5%	93.1%	1, 2	Yes
Three DAP items: Pregnant: good thing for me, Baby: end of the world for me, Baby: makes me smile.	68%	1.0%	1.49	69.4%	84.4%	0.77	46.3%	93.5%	1, 2	Yes
Three DAP items: Pregnant: good thing for me, Baby: want, Baby: end of the world for me.	72%	13.9%	1.49	66.7%	90.3%	0.79	57.1%	93.3%	1	Yes
Three DAP items: Pregnant: good thing for me, Baby: end of the world for me, Baby: hard for me to manage.	76%	8.8%	1.82	75.0%	77.2%	0.76	38.8%	94.1%	1, 3	Yes
Four DAP items: Pregnant: good thing for me, Pregnant: excited, Baby: want, Baby: end of the world for me.	73%	1.4%	1.63	66.7%	89.2%	0.78	54.5%	93.2%	1, 2	Yes
Four DAP items: Pregnant: good thing for me, Pregnant: excited, Baby: end of the world for me, Baby: hard to achieve other things.	81%	1.2%	1.88	66.7%	83.9%	0.75	44.4%	92.9%	All	Yes
Six DAP items: Pregnant: wouldn't mind, Pregnant: unhappy, Baby: want, Baby: positive addition to life, Baby: makes me smile, Baby: hard for me to manage.	73%	1.6%	1.93	75.0%	80.9%	0.78	43.2%	94.4%	All	Yes

AUROC, area under the receiver operating curve; DAP, Desire to Avoid Pregnancy; NPV, negative predictive value; PPV, positive predictive value.

Adding a second item of ‘Baby: end of the world for me’ improved the specificity and PPV, without affecting the AUROC. The highest AUROC (0.79) was achieved with the combination of three items ‘Pregnancy: good thing for me’, ‘Baby: want’ and ‘Baby: end of the world for me’, which also had the highest PPV (57.1%). The item combinations had AUROCs between 0.77 and 0.79, in line with that of the individual item ‘Pregnancy: good thing for me’ suggesting little additional gain.

## Discussion

This is the first examination of the predictive ability of two measures (DAP and APPS) and three single questions (‘feeling’, ‘trying’ and ‘thinking’) about pregnancy preferences on a sample that is broadly representative of women of reproductive age in the UK. In terms of the AUROC and the predicted probability of pregnancy in the next year, the performance of the APPS and the other questions was weaker than the complete DAP, though to varying degrees. The APPS and the other questions are all shorter than the DAP and therefore potentially less burdensome for clinical use. Arguably, their poorer performance is offset by their brevity. Our data show that the single question ‘Are you currently trying to get pregnant?’ had low sensitivity (37%) and the lowest AUROC (0.67). While women answering yes to this question were highly likely to become pregnant (PPV 69%) there were more pregnancies among women who answered no. Questions like this, which force women to answer yes-or-no, fail to recognise the complexity of the concept of pregnancy preferences, leading to misclassification.^32^

When considering which question(s) might be best for clinical use, the trade-off between sensitivity and specificity, and the PPV and NPV is important. In addition, the number of questions that need to be asked, and the complexity of combining them to achieve a score, could be a barrier in consultations, therefore, having fewer questions will likely increase uptake. As our analysis shows, single questions generally have lower AUROCs than a set of questions, however, selected items from the DAP, which have been developed based on rigorous theoretical groundwork, do have higher AUROCs than the less carefully constructed questions. Neither the DAP nor the APPS were designed to be spoken, whereas the other questions lend themselves more easily to being asked verbally.

While we did not use the OKQ, good correlation was previously seen between the DAP score and the OKQ in the USA.^33^ As that study noted, DAP scores ranged widely within each OKQ response option; women who responded to the OKQ with ‘want to get pregnant in future but not in next year’ had DAP scores ranging from 1 to 4. Our data demonstrated the same patterns; women who reported feeling ‘mostly negative’ towards pregnancy had DAP scores ranging from 0.286 to 4, this shows the nuance that the DAP can capture and demonstrates the heterogeneity missed by a single question. In addition, the OKQ is frequently the least-favoured option in studies asking women and healthcare professionals’ preference from a range of questions.^5 34 35^ There have been no comparisons made to the APPS.

There are well-known barriers to implementation of preconception care,^36^ and it is likely that these apply to pregnancy intention screening as the gateway to identifying who requires preconception care. Lack of time is a commonly cited barrier,^37^ as is the lack of clarity over whose role it should be.^36^ However, there is growing evidence of the feasibility and acceptability, to women and healthcare professionals, of pregnancy intention screening,^5–7 38^ and of the effectiveness of preconception interventions in primary care.^39^

### Strengths and limitations

We used a large, broadly representative dataset and a validated measure (the DAP) to assess the performance of a range of questions for assessing pregnancy preferences, and have provided preliminary evidence that the APPS is valid in the UK. Given the intellectual property limitations on the use of the OKQ, which affect the feasibility of national rollout in the UK, we did not include it in our research. While we used a split dataset to conduct cross-validation within our sample, which is a strength, confirmation of our findings in other populations, particularly those that are more diverse in terms of ethnicity, education and socioeconomic status, would provide further confidence in selecting the most appropriate question(s). The selected DAP items/combinations were based on their potential as screening tools for pregnancy, as well as theoretical considerations; item-response theory-based analysis of the psychometric properties of these combinations could be conducted.

## Conclusion

Discussions about pregnancy preferences are important, regardless of whether the woman wants to become pregnant in the future or not. Ensuring that those who do not wish to become pregnant have the right support to avoid pregnancy is just as important as identifying those who might benefit from prepregnancy health advice or those who may benefit from both. Equally, for those who have never formally considered their preferences, it provides an opportunity to empower them and increase their agency by highlighting that people do have choices about pregnancy and parenthood (recognising the effect of external factors) and encouraging them to explore their aspirations.

In the context of a face-to-face clinical encounter, where the full DAP is less likely to be suitable, a tool to assess people’s preferences regarding a future pregnancy needs to be both practical (short) and discriminative, that is, identify who is and is not likely to become pregnant in the short term so that the appropriate advice can be given. Based on our findings, we recommend exploring the acceptability to women and healthcare professionals of a single item from the DAP (‘It would be a good thing for me if I became pregnant in the next 3 months’) or a combination of this with two additional DAP items (either ‘Baby: want’ and ‘Baby: end of the world for me’ or ‘Pregnant: excited’ and ‘Baby: hard to achieve other things’) adapted from the written format to a spoken one.

## Data Availability

Data are available in a public, open access repository. The dataset is available in the UCL Research Data Repository, doi 10.5522/04/21263007.
